# Pioglitazone Protects Against Renal Ischemia-Reperfusion Injury via the AMP-Activated Protein Kinase-Regulated Autophagy Pathway

**DOI:** 10.3389/fphar.2018.00851

**Published:** 2018-08-06

**Authors:** Wenlin Chen, Xiaoqing Xi, Shuangyang Zhang, Cong Zou, Renrui Kuang, Zhenfeng Ye, Yawei Huang, Honglin Hu

**Affiliations:** ^1^Department of Urology, The Second Affiliated Hospital of Nanchang University, Nanchang, China; ^2^Department of Urology, The Hospital of Wuchang, Wuhan, China; ^3^Department of Endocrinology, The Fourth Affiliated Hospital of Nanchang University, Nanchang, China

**Keywords:** pioglitazone, renal ischemia reperfusion injury, peroxisome proliferators-activated receptor-γ, autophagy, cell apoptosis

## Abstract

Renal ischemia-reperfusion injury (IRI) is a major cause of acute renal failure. Our previous studies have shown that pioglitazone, a peroxisome proliferators-activated receptor (PPAR)-γ agonist used in type 2 diabetes, protects against renal IRI; however, the molecular mechanism underlying the renoprotective effects of pioglitazone is still unclear. In this study, we investigated the role of AMP-activated protein kinase (AMPK)-regulated autophagy in renoprotection by pioglitazone in IRI. To investigate whether pioglitazone protects renal cells from IRI, an *in vivo* renal IRI model was used. Cell apoptosis in the kidneys was determined by TUNEL staining. Western blotting was used to determine the expression of AMPK, autophagy-related proteins, and caspase-3/8 proteins in the kidneys. In a rat model of IRI, pioglitazone decreased the increased serum creatinine and urea nitrogen, improved renal histological score, and decreased the cell injury. Pioglitazone also increased AMPK phosphorylation, inhibited p62 and cleaved caspase-3/8 proteins, and activated autophagy-related proteins LC3 II and Beclin-1 in the kidneys of IRI rats. Moreover, GW9662, as a selective inhibitor of PPAR-γ, inhibited the protective effects of pioglitazone. These results suggest that pioglitazone exerts its protective effects in renal IRI via activation of an AMPK-regulated autophagy signaling pathway.

## Introduction

Ischemia reperfusion injury (IRI) is a pathological state caused by the recovery of blood flow after tissue and organ ischemia, resulting in serious cell dysfunction and destruction of tissue structure (Roberts et al., 2013; Messner et al., 2016). Since the kidney has high perfusion, severe IRI could occur after urologic surgical procedures, such as renal transplantation and partial nephrectomy (Rodriguez et al., 2013; Malek and Nematbakhsh, 2015). Although various signaling mechanisms are currently under investigation as potential targets limiting cell injury due to such ischemia events, including the release of ROS, cell apoptosis, necrosis, infiltration by inflammatory cells, and the release of active mediators leading to tissue damage (Friedewald and Rabb, 2004; Kieran and Rabb, 2004), the molecular mechanisms responsible for renal IRI remain largely unknown.

Autophagy is a physiological and pathological process in which cells use lysosomes to degrade their own damaged, aging organelles and macromolecules to maintain cell stability (Go et al., 2015). Autophagy has been reported to play a protective role in IRI, indicating that enhanced autophagy is beneficial for the improvement of IRI (Ling et al., 2016).

Pioglitazone hydrochloride (Pio) is a thiazolidine two-ketone drug. As a peroxisome proliferators-activated receptor-γ (PPAR-γ) agonist, it has been used in the treatment of type 2 diabetes. Recent studies have shown that Pio can be used to protect the kidneys, myocardium, and brain against IRI (Ahmed et al., 2011; Zhang et al., 2011; Zhang X. Y. et al., 2013; Zou et al., 2013). Morrison et al. (2011) reported that acute treatment with rosiglitazone, a PPAR-γ agonist, can reduce ischemic injury in a nondiabetic mouse heart via modulation of the AMP-activated protein kinase (AMPK) signaling pathway. In this study, we hypothesized that Pio could activate the signals of AMPK and autophagy to attenuate renal IRI. We, therefore, investigated the roles of AMPK and autophagy in the renoprotective effects of Pio using a rat model for renal IRI.

## Materials and methods

### Experimental animals

One-hundred and twenty male Sprague–Dawley (SD) rats (200–250 g, and specific pathogen-free) were purchased from Beijing Vital River Laboratory Animal Technology Co., Ltd. This study was carried out in compliance with the protocols approved by the Animal Care and Use Committee at Nanchang University (China).

### Materials and reagents

GW9662 and 3-MA were purchased from Sigma (USA). Rabbit antibodies against PPAR-γ, LC-3I, LC-3II, Beclin-1, AMPK, P62, and caspase-3/8 and a polyclonal antibody against β-actin were purchased from Abcam (USA). A terminal deoxynucleotide transferase-mediated dUTP nick-end labeling (TUNEL) detection kit was purchased from Wuhan Boster Biological Technology Co. Ltd. Kits for the extraction of TagDNA and RNA were obtained from Cwbiotech (Beijing, China); Kim-1 and NGAL ELISA kits were purchased from USCN Life Science Inc. (Wuhan, China).

### Experimental design

The SD rats were randomly divided into 12 groups: 1) PBS+IRI (*n* = 10); 2) Pio+IRI (*n* = 10); 3) Pio+GW9662+IRI (*n* = 10); 4) GW9662+IRI (*n* = 10); 5) Pio+3-MA+IRI (*n* = 10); 6) 3-MA+IRI (*n* = 10); 7) PBS+sham (*n* = 10); 8) Pio+sham (*n* = 10); 9) Pio+GW9662+sham (*n* = 10); 10) GW9662+sham (*n* = 10); 11) Pio+3-MA+ sham (*n* = 10); 12) 3-MA+sham (*n* = 10). Pio dissolved in PBS was intraperitoneally injected (10 mg/kg/day), once a day, for 3 days before inducing renal ischemia in rats. GW9662, as a selective inhibitor of PPAR-γ, was intraperitoneally injected 4 h before inducing renal ischemia in rats (1 mg/kg bodyweight). 3-MA, as an inhibitor of autophagy, was also intraperitoneally injected 4 h before renal ischemia in rats (5 mg/kg bodyweight).

### Rat model of renal IRI

The rats were fasted 8–12 h before operation and were anesthetized by an intraperitoneal injection of 2% chloralic hydras (2 mL/100 g weight). The detailed procedure is described in our previous reports (Hu et al., 2012; Zou et al., 2013). Briefly, a 1.5–2-cm incision was made along the median abdominal line. The skin and peritoneum were separated layer by layer to reach the abdominal cavity. The intestine was pushed to one side. Then, the renal pedicles were exposed and clamped bilaterally for 45 min. For reperfusion, the clamp was removed and the kidneys monitored for color change to confirm blood reflow before suturing the incision. During the operation, saline should be provided to keep the rats fully hydrated. After operation, the abdominal cavity was closed by layered suture. The rats were kept warm at 24~29°C with water and food provided *ad libitum*. For the sham groups, animals underwent the same procedure except for renal pedicle clamping.

### Measurement of renal function

Blood samples (0.5 mL) were obtained through orbital canthus plexus 24 h after reperfusion and then centrifuged (4,500 r/min for 10 min). Serum was extracted and the level of creatinine and urea nitrogen was measured.

### Histopathologic evaluation of the kidney

The rats in different groups were sacrificed 24 h after reperfusion with the kidneys taken out. The kidneys were cut coronally, fixed in 10% buffered formalin, and embedded in paraffin. Four-micrometer sections were prepared and stained with hematoxylin and eosin (H&E). Renal tubular necrosis was observed under an optical microscope. Each kidney slice was randomly selected to observe 20 visual fields at the cortico-medullary area. A semi-quantitative pathological assessment was performed to grade the degree of renal tubular necrosis. Higher scores represented more severe damage (maximum score = 4): 0 = normal kidney; 1 = minimal necrosis, < 5% involvement; 2 = mild necrosis, 5–25% involvement; 3 = moderate necrosis, 25–75% involvement; and 4 = severe, >75% involvement.

### Assessment of renal Kim-1 and NGAL

The rats in different groups were sacrificed 24 h after reperfusion with the kidneys taken out. Renal Kim-1 and NGAL were measured in tissue homogenates using specific ELISA kits according to the manufacturer's protocols.

### TUNEL assay

A TUNEL staining assay was used to detect DNA strand-breaks. Fixed kidney sections obtained 24 h after renal IRI were deparaffinized in xylene and rehydrated through a graded ethanol series. The number of TUNEL-positive nuclei per 200 × field was evaluated in 25 fields per section.

### Reverse transcription polymerase chain reaction (RT-PCR)

The rats in different groups were sacrificed 24 h after reperfusion with the kidneys taken out. Total RNA was extracted from rat kidney using Trizol reagent (Cwbiotech, Beijing, China) according to the manufacturer's instructions. Reverse transcription into cDNA was performed using a TaKaRa RNA polymerase chain reaction (PCR) kit Version 3.0 (TaKaRa, Dalian, China) for PCR analysis; primers were designed. RT-PCR was performed with the SYBR Premix Ex Taq kit (Takara, Dalian, China) for fluorescence detection during amplification on an ABI 7500 Fast Real-Time PCR System (Applied Biosystems, USA). PCR cycling was performed under the following conditions: initial denaturation at 95°C for 30 s and 40 thermal cycles of 95°C for 5 s, 60°C for 34 s, and 72°C for 30 s.

### Western blot

The rats in different groups were sacrificed 24 h after reperfusion with the kidneys taken out. The kidneys were homogenized in ice-cold buffer [1 mM Tris–HCl buffer, pH 7.5, with a cocktail of protease inhibitors, 25 mM NaF, 10 mM NaV, 0.5 mol/l EDTA, and 1% Triton X-100] and then centrifuged at 20,000 g for 20 min. Protein concentration was determined using the Bradford protein assay. Aliquots of 200 μg of protein extracts were separated on 10–15% SDS-polyacrylamide gels and transferred to nitrocellulose membranes. Membranes were blocked with 5% milk in TBST buffer (10 mM Tris-base, 100 mM NaCl, 0.1% Tween 20, pH 8.0) and then probed overnight at 4°C with primary antibodies. The membranes were then incubated with 1:1,000 goat anti-mouse horseradish peroxidase-conjugated secondary antibodies. Protein bands were detected using Super Signal West Pico Chemiluminescent Substrate. Images of blots were acquired for quantification using a digital imager and analyzed with imaging software.

### Statistical analysis

All data were analyzed using GraphPad Prism5.0, represented as means ± S.E. Data were analyzed with one-way ANOVA plus the Tukey *post-hoc* multiple-comparisons test for comparison of mean values among multiple groups. A P value of less than 0.05 was accepted as statistically significant.

## Results

### Pio decreased the levels of serum creatinine and urea nitrogen

**Figure 1:**
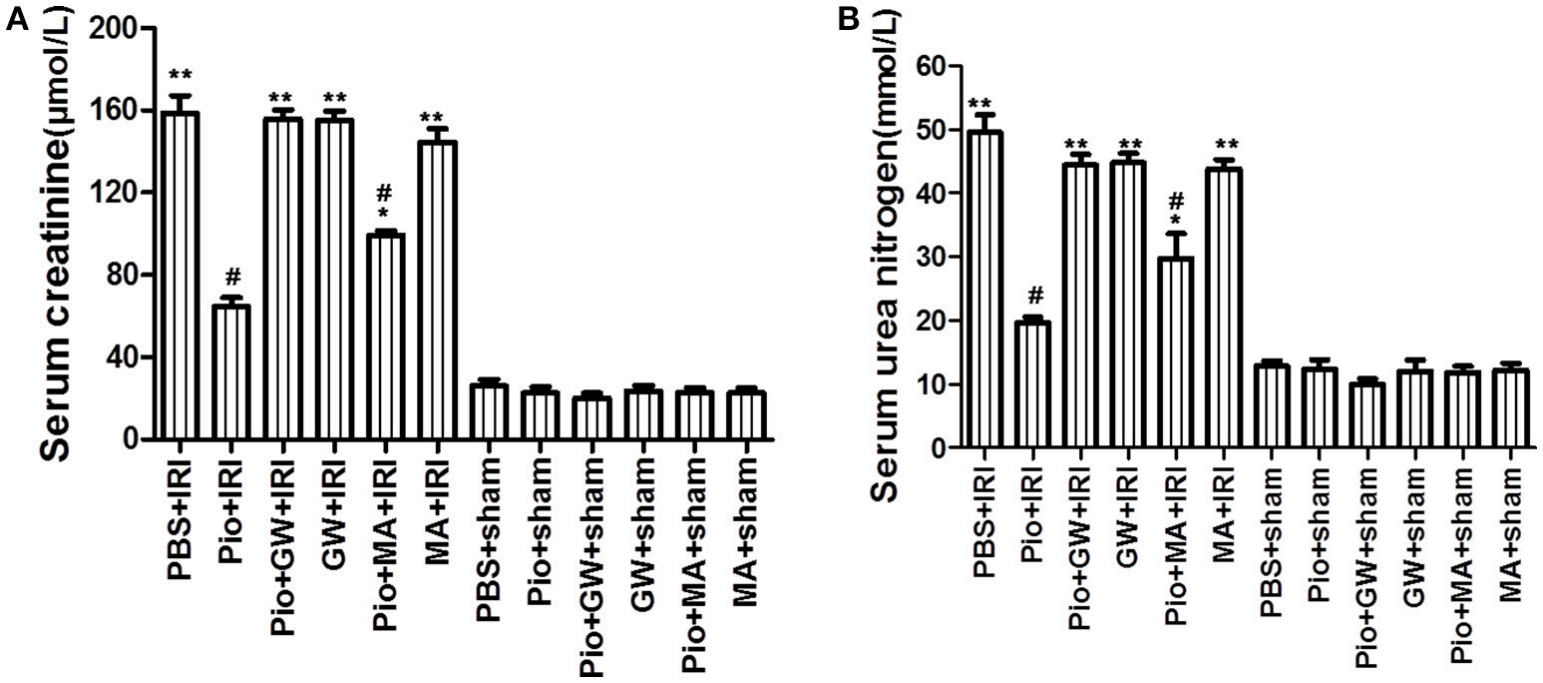
Pioglitazone protects against renal IRI. **(A)** Serum creatinine in different groups at 24 h after renal IRI. **(B)** Serum urea nitrogen in different groups at 24 h after renal IRI. Data shown are means ± SE. Compared with the PBS+sham group, ^*^P < 0.05, ^**^P < 0.01. Compared with the PBS+IRI group, ^#^*P* < 0.05. *n* = 10 in each group.

The serum creatinine and urea nitrogen levels in the PBS+IRI, Pio+GW+IRI, GW+IRI, Pio+MA+IRI, and MA+IRI groups were significantly higher than those in the sham group (*P* < 0.05); however, the administration of Pio markedly inhibited the increase. Serum creatinine and urea nitrogen levels in the Pio+IRI and Pio+MA+IRI groups were significantly lower than those in the PBS+IRI group (*P* < 0.05); however, these parameters showed no significant improvement in the Pio+GW+IRI, GW+IRI, and MA+IRI groups (*P* > 0.05) (Figure 1). These data suggested that Pio protected renal function from the effects of IRI. This protective effect could be inhibited by GW9662, but not 3-MA.

### Pio decreased apoptotic cell death in the kidney

**Figure 2:**
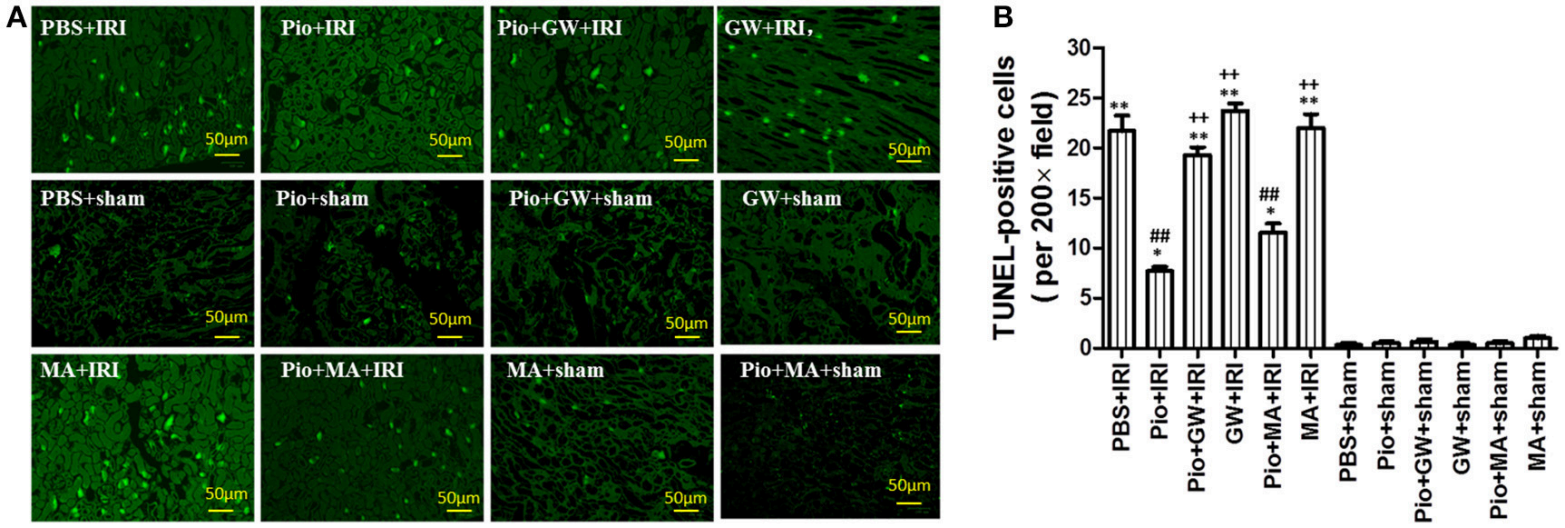
Pioglitazone decreases cell apoptosis at 24 h after renal IRI. **(A)** Representative photographs of kidney sections stained for apoptotic cells (green). Scale bar: 50 μm. Original magnification: 200x. **(B)** Quantitative analysis of TUNEL-positive cells in the kidneys. Compared with the PBS+sham group, ^*^represents for *P* < 0.05, ^**^represents for *P* < 0.01; Compared with the PBS+IRI group, ^##^represents for *P* < 0.01; Compared with the Pio+IRI group, ^++^represents for *P* < 0.01. *n* = 6 in each group. TUNEL, terminal transferase dUTP nick-end labeling.

The apoptosis rates in the other IRI groups were significantly higher than those in the PBS+sham group (*P* < 0.05). However, there was no significant difference between the PBS+sham and other sham groups (*P* > 0.05). The apoptosis rates in the Pio+IRI and Pio+MA+IRI groups were significantly lower than those in the PBS+IRI group (*P* < 0.05). In comparison with those in the Pio+IRI group, the apoptosis rates in Pio+GW+IRI, GW+IRI, and MA+IRI groups significantly increased (*P* < 0.05). The above results collectively suggest that Pio played an important role in suppressing the apoptosis of renal cells in IRI rat models and GW9662 inhibited the effects of Pio (Figures 2A,B).

### Pio decreased tubular damage

**Figure 3:**
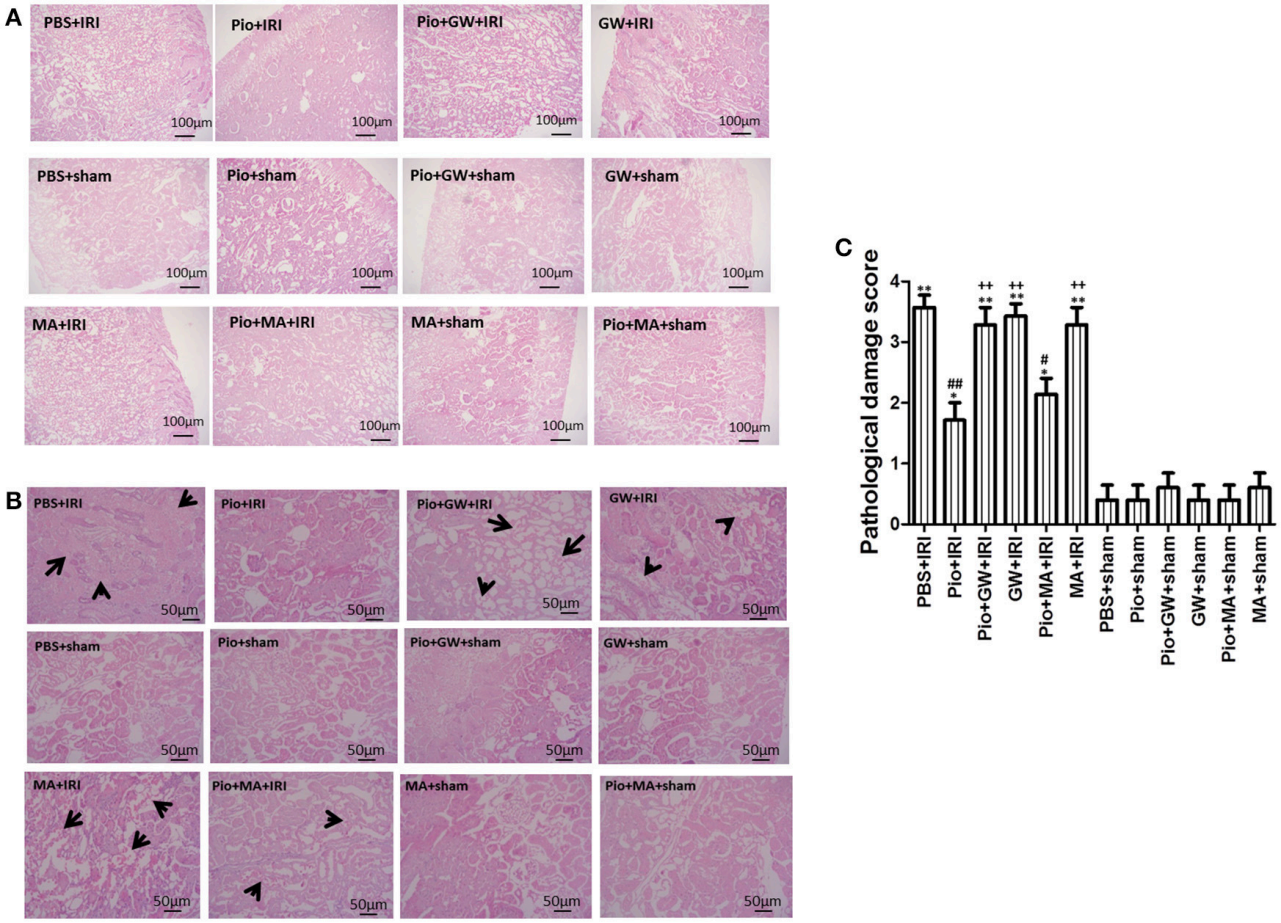
Histological evaluation of renal tissue at 24 h after renal IRI. **(A)** (original magnification 100x), **(B)** (original magnification 200x) Representative photographs of kidney sections stained with hematoxylin and eosin. **(C)** Histological scores in kidney sections. Arrows indicate tubular dilatation, swelling, congestion, vacuolization and tubular necrosis in the kidneys. Compared with the PBS+sham group, ^*^represents for *P* < 0.05, ^**^represents for *P* < 0.01; Compared with the PBS+IRI group, ^#^represents for *P* < 0.05, ^##^represents for *P* < 0.01; Compared with the Pio+IRI group, ^++^represents for *P* < 0.01. *n* = 6 in each group.

In all sham-operation groups, renal tissue sections had a normal morphology (Figures 3A,B). Histological examination of the kidneys exposed to PBS+IRI, Pio+GW+IRI, GW+IRI, MA+IRI, and Pio+MA+IRI showed the distinctive pattern of ischemic renal injury, which included widespread degeneration of tubular architecture, tubular dilatation, swelling, congestion, vacuolization, and tubular necrosis (Figures 3A,B). However, Pio alleviated the tubular structure derangement and tubular necrosis that were observed at 24 h after reperfusion following ischemia (Figures 3A,B). The pathological scores of renal tubule injury in the other IRI groups were significantly higher than those in the PBS+sham group (*P* < 0.05). However, there was no significant difference between the PBS+sham and other sham groups (*P* > 0.05). The pathological scores of renal tubule injury in the Pio+IRI and Pio+MA+IRI groups were significantly lower than those in the PBS+IRI group (*P* < 0.05). The pathological scores of renal tubule injury in the Pio+GW+IRI, GW+IRI, and MA+IRI groups were significantly higher than those in the Pio+IRI group (*P* < 0.05) (Figure 3C).

**Figure 4:**
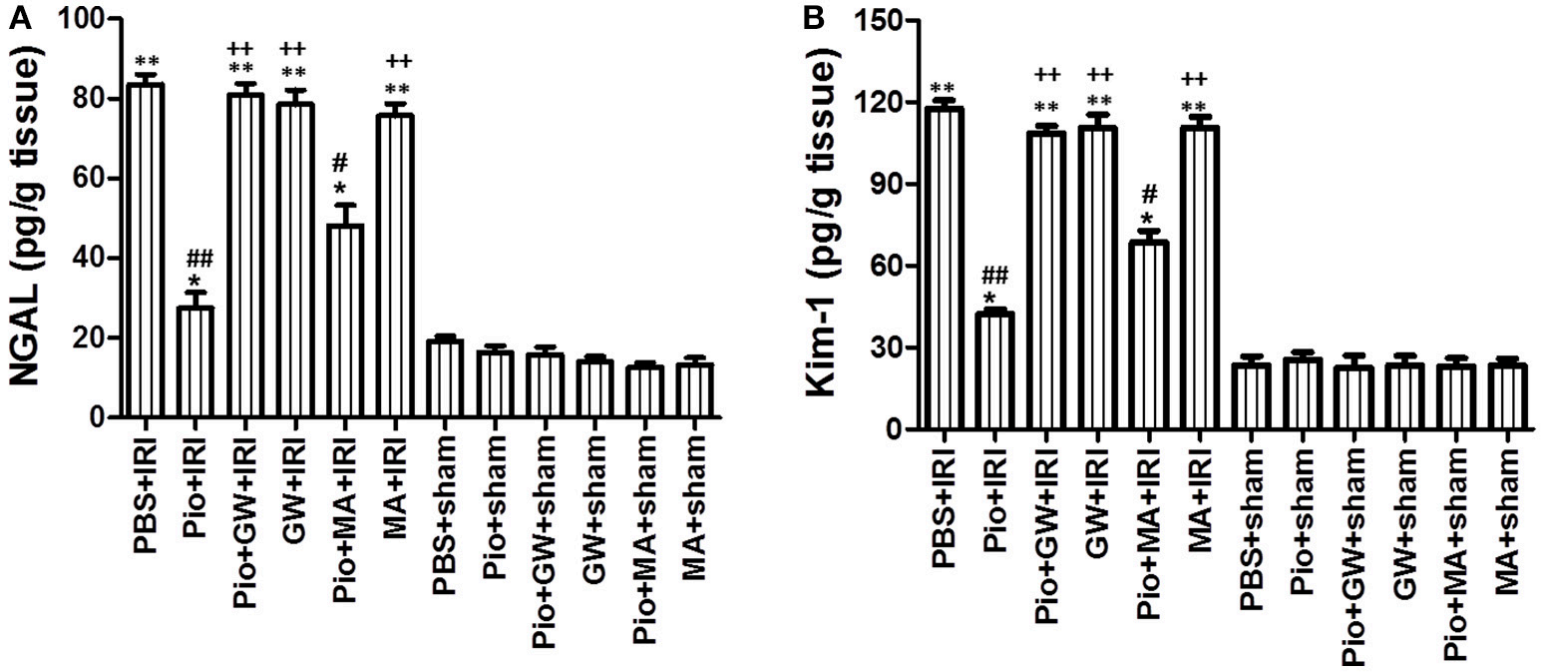
Pioglitazone decreases renal NGAL and Kim-1. **(A)** Renal NGAL in different groups at 24 h after renal IRI. **(B)** Renal Kim-1 in different groups at 24 h after renal IRI. Data shown are means ± SE. Compared with the PBS+sham group, ^*^represents for *P* < 0.05, ^**^represents for *P* < 0.01; Compared with the PBS+IRI group, ^#^represents for *P* < 0.01, ^##^represents for *P* < 0.01; Compared with the Pio+IRI group, ^++^represents for *P* < 0.01. *n* = 6 in each group. NGAL, neutrophil gelatinase-associated lipocalin. Kim-1, kidney injury molecule-1.

The same pattern was also observed for the content of other specific kidney injury markers, NGAL and Kim-1, as depicted in Figure 4. The level of these parameters was higher in the injured nontreated kidneys than in the sham group. Nevertheless, Pio could significantly decrease the levels of NGAL and Kim-1 in the kidneys subjected to IRI; however, GW9662 and 3-MA could suppress this effect (Figure 4).

### Pio increased the PPAR-γ activity in renal tissue

**Figure 5:**
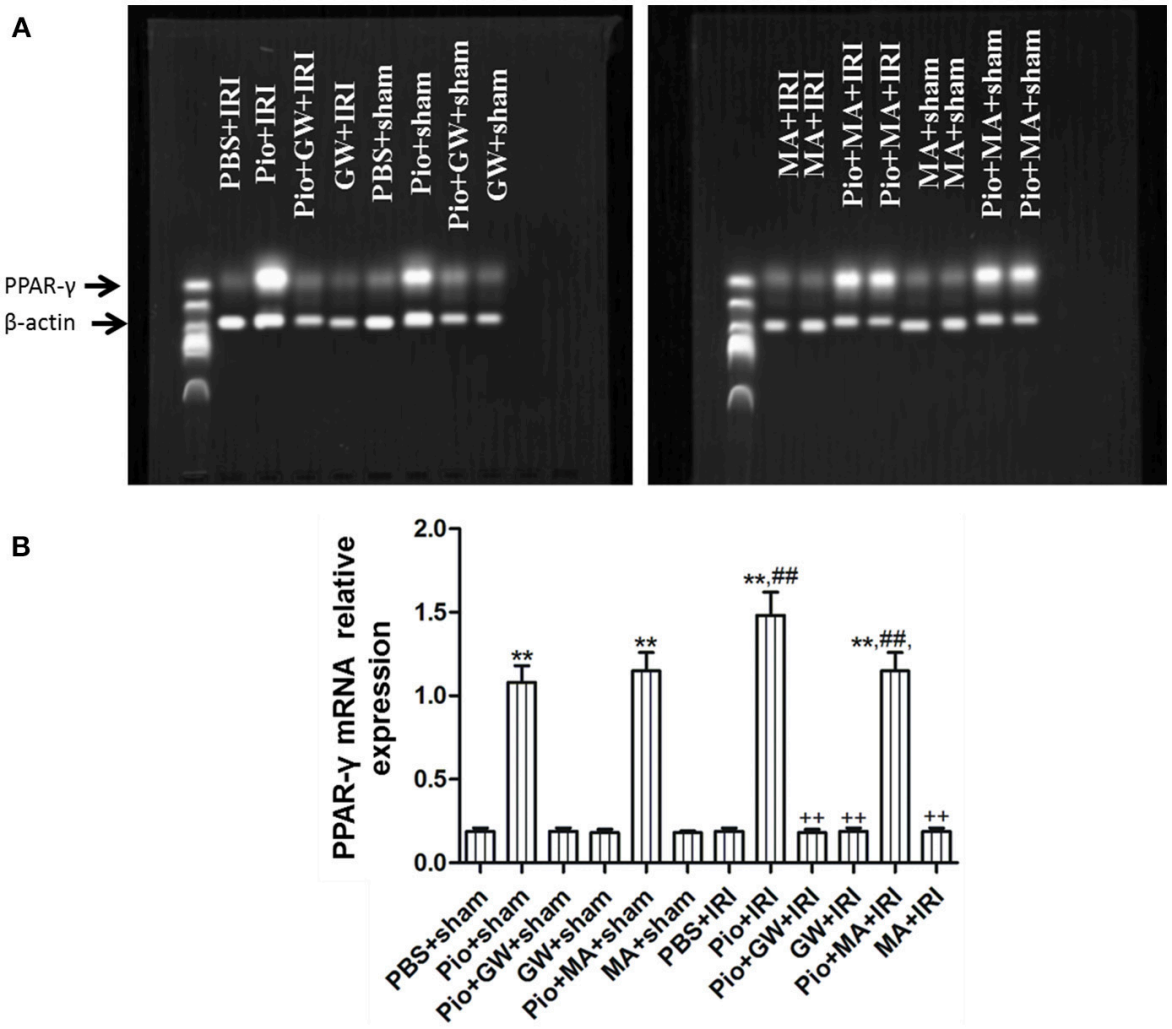
Pioglitazone up-regulates PPAR-γ mRNA. **(A)** Representative bands show PPAR-γ mRNA in the kidneys at 24 h after renal IRI. **(B)** Quantitative analysis of PPAR-γ mRNA in the kidneys at 24 h after renal IRI. Compared with the PBS+sham group, ^**^represents for *P* < 0.01; Compared with the PBS+IRI group, ^##^represents for *P* < 0.01; Compared with the Pio+IRI group, ^++^represents for *P* < 0.01. *n* = 6 in each group. PPAR-γ, peroxisome proliferators-activated receptor-γ.

There was no significant difference in the expression of PPAR-γ mRNA among the PBS+IRI, Pio+GW+IRI, GW+IRI, Pio+GW+sham, GW+sham, and MA+sham groups (*P* > 0.05), compared with that in the PBS+sham group. In contrast, the expression of PPAR-γ mRNA in the Pio+IRI, Pio+sham, Pio+MA+IRI, and Pio+MA+sham groups was significantly up-regulated (*P* < 0.05). The expression of PPAR-γ mRNA in the Pio+IRI and Pio+MA+IRI groups was higher than that in the PBS+IRI group (*P* < 0.05). However, the expression of PPAR-γ mRNA in the Pio+GW+IRI, GW+IRI, and MA+IRI groups was remarkably down-regulated compared with that in the Pio+IRI group (*P* < 0.05). These results reveal the role of Pio in promoting PPAR-γ mRNA expression in IRI rat models, while GW9662 inhibited the effects of Pio on PPAR- γ mRNA expression (Figure 5).

**Figure 6:**
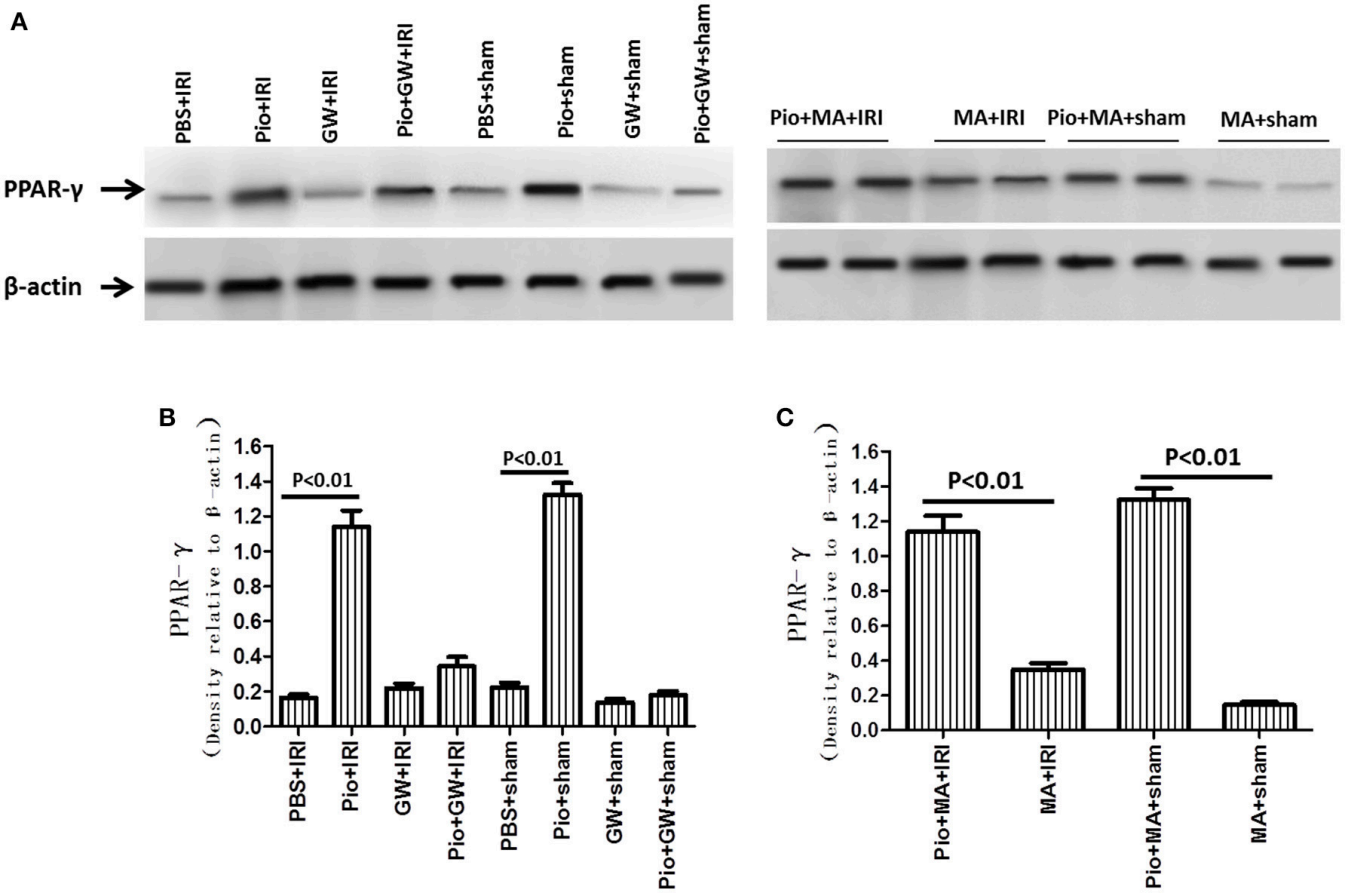
Pioglitazone increases PPAR-γ protein expression. **(A)** Representative western blots show PPAR-γ protein level in the kidneys at 24 h after renal IRI. **(B,C)** Quantitative analysis of PPAR-γ protein level in the kidneys. Values are expressed as means ± SE. *n* = 6 in each group. PPAR-γ, peroxisome proliferators-activated receptor-γ.

The expression of PPAR -γ protein in the Pio+sham and Pio+GW+sham groups was higher than that in the PBS+sham group (*P* < 0.05). The expression of PPAR-γ protein in the Pio+IRI and Pio+GW+IRI groups was significantly higher than that in the PBS+IRI group (*P* < 0.05). GW9662 inhibited the expression but 3-MA could not. The above results together reveal that Pio played an important role in promoting the expression of PPAR-γ protein in renal cells in IRI rat models (Figure 6).

### Pio increased the expression of LC3 II and Beclin-1 in renal tissue

**Figure 7:**
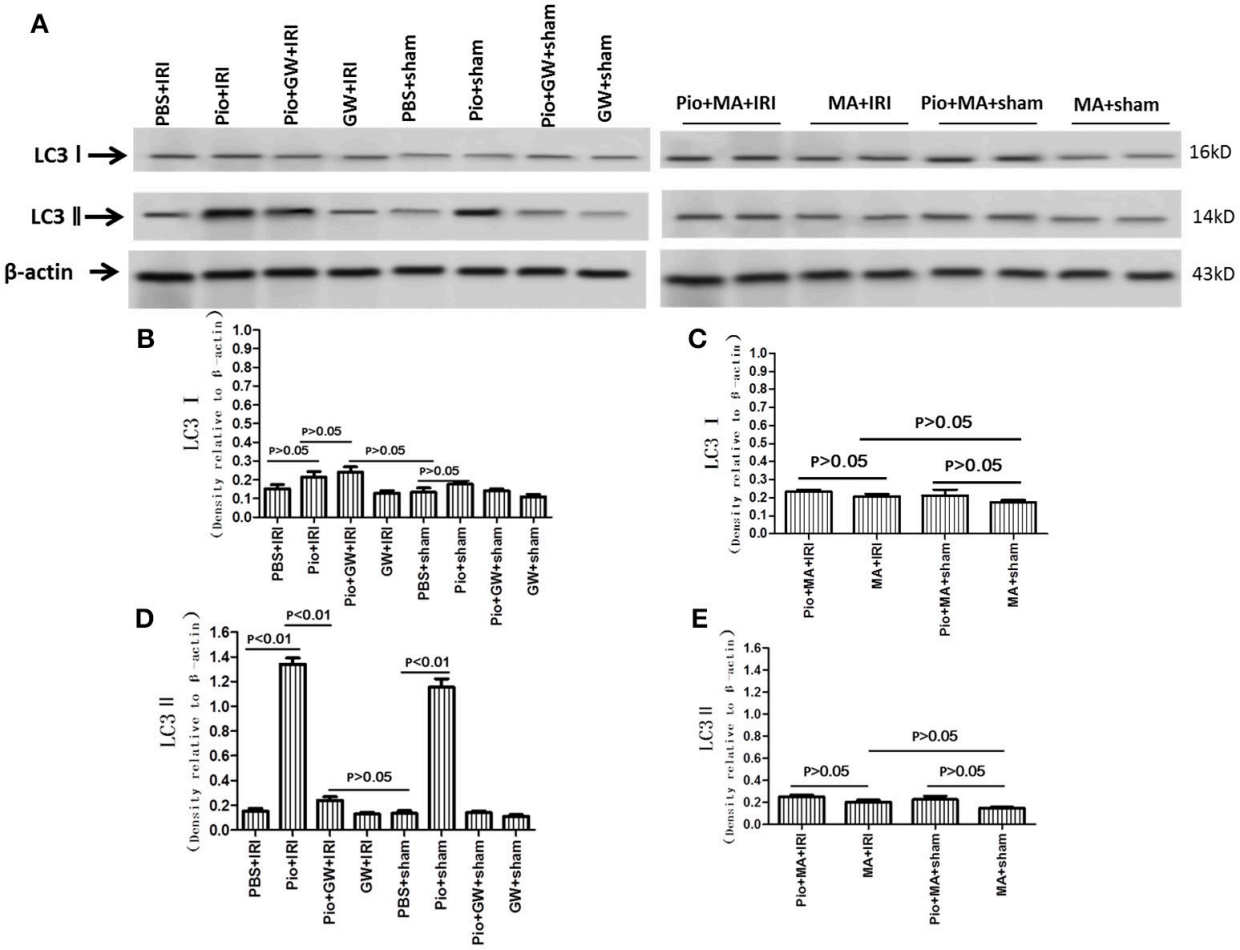
Expression of LC3 I and LC3 II proteins at 24 h after renal IRI. **(A)** Representative western blots show LC3 I and LC3 II protein levels in the kidneys. **(B–E)** Quantitative analysis of LC3 I and LC3 II protein levels in the kidneys. Values are expressed as means ± SE **(B–E)**. *n* = 6 in each group.

**Figure 8:**
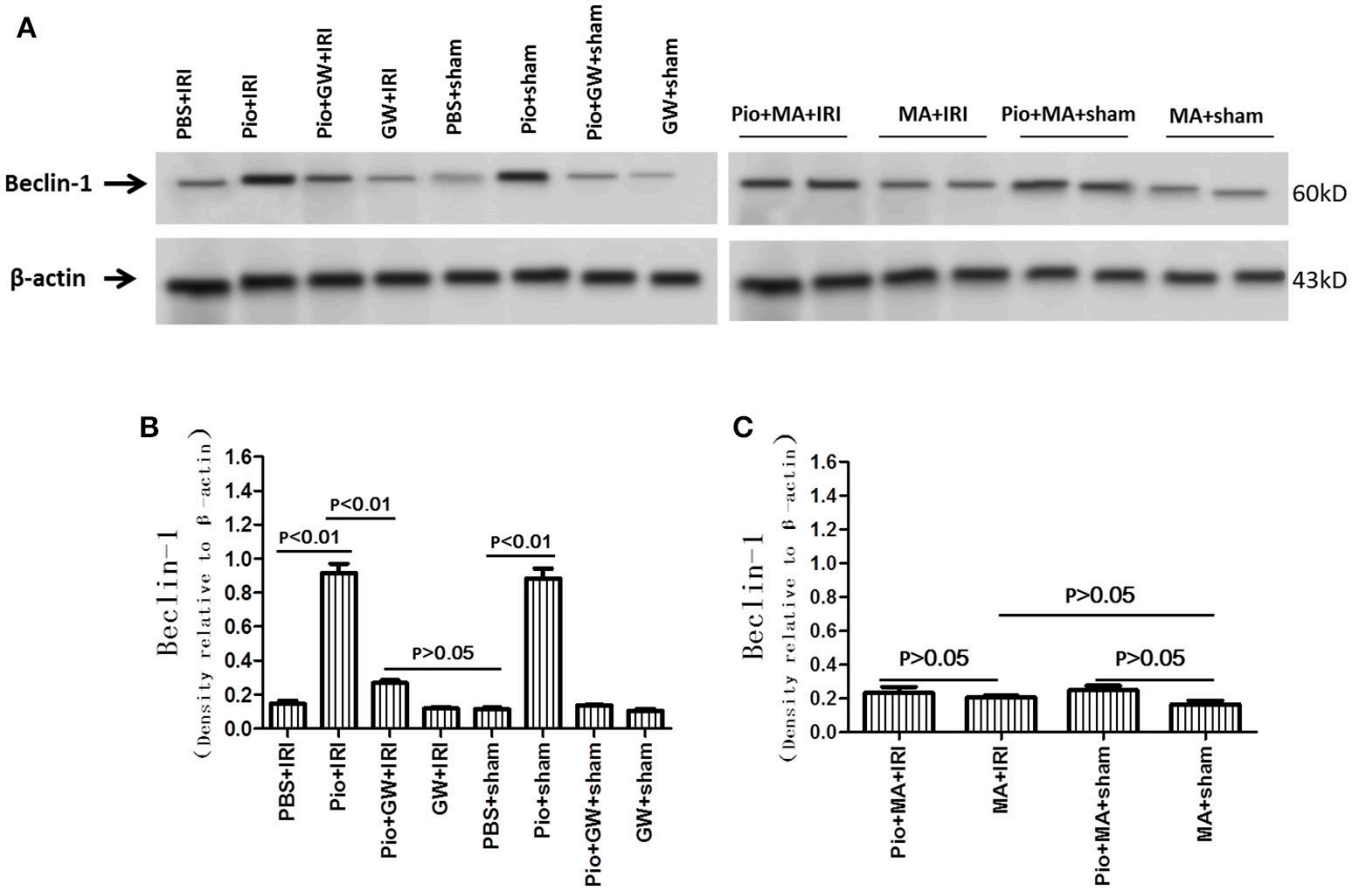
Pioglitazone increases Beclin-1 protein at 24 h after renal IRI. **(A)** Representative western blots show Beclin-1 protein level in the kidneys. **(B,C)** Quantitative analysis of Beclin-1 protein level in the kidneys. Values are expressed as means ± SE **(B,C)**. *n* = 6 in each group.

The expression of LC3 II and Beclin-1 protein in the Pio+ sham group was significantly higher than that in the PBS+sham group (*P* < 0.05). Similarly, the expression of LC3 II and Beclin-1 protein in the Pio+IRI group was higher than that in the PBS+IRI group (*P* < 0.05). However, in the Pio+GW+IRI groups, the protein expression was inhibited significantly. As shown in Figures 7, 8, 3-MA could suppress the protein expression of LC3 II and Beclin-1. Therefore, Pio played an important role in promoting the protein expression of LC3 II and Beclin-1 in renal cells in IRI rat models, while GW9662 and 3-MA inhibited the expression, with 3-MA showing a more pronounced effect. However, Pio had no effect on the expression of LC3 I (Figures 7, 8).

### Pio decreased the expression of P62 in renal tissue

**Figure 9:**
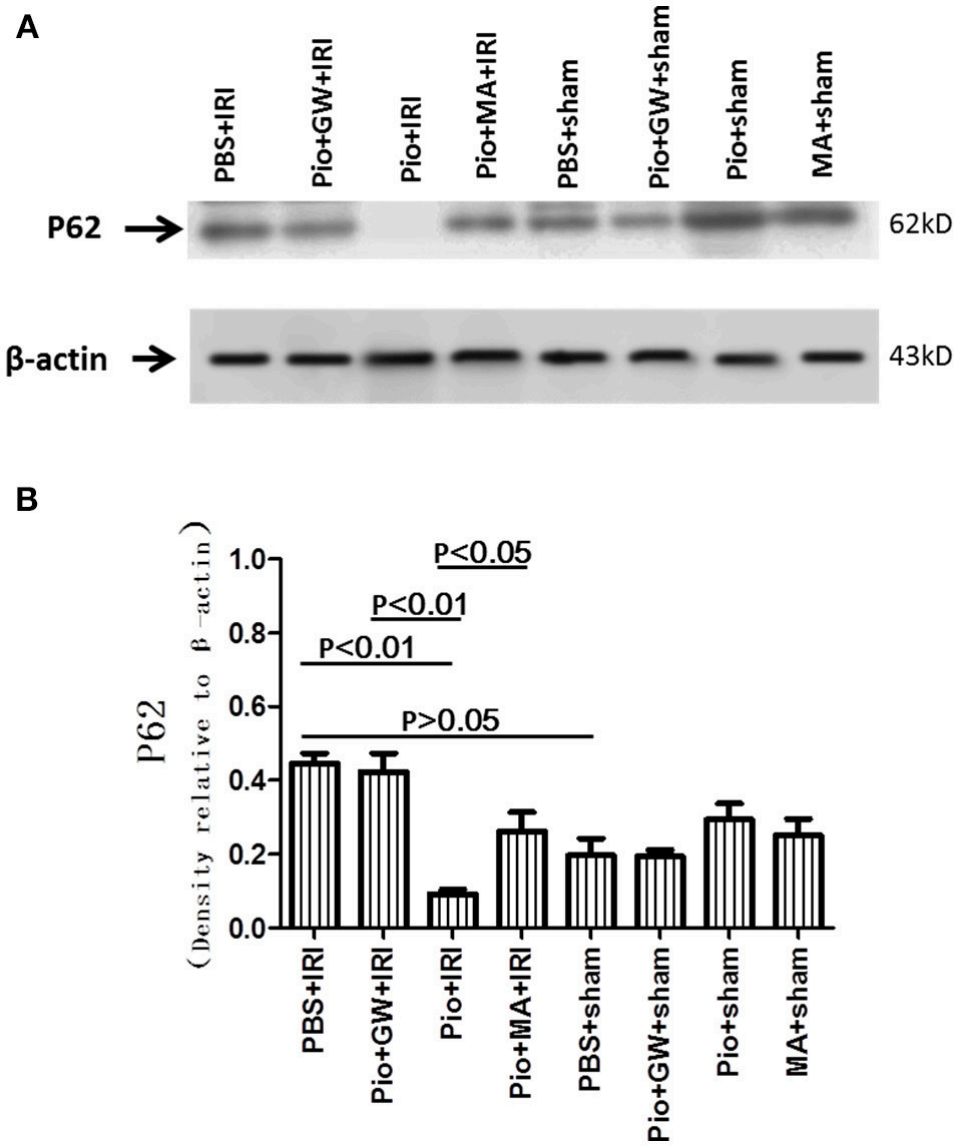
Pioglitazone decreases p62 protein in renal tissue at 24 h after renal IRI. **(A)** Representative western blots show p62 protein level in the kidneys. **(B)** Quantitative analysis of p62 protein level in the kidneys. Values are expressed as means ± SE **(B)**. *n* = 6 in each group.

Next, the level of p62 was examined. p62, an autophagic substrate, is widely used as an indicator because it is involved in the dynamic process of the delivery of autophagic substrates to the lysosome and degradation of autophagic substrates inside the lysosome. Our results revealed no difference in the expression of P62 in all the sham-operation groups. The level of p62 decreased in the Pio+IRI group, which was consistent with the autophagy induced by Pio. GW9662 and 3-MA inhibited this effect (Figure 9).

### Pio increased the expression of phosphorylated AMPK in renal tissue

**Figure 10:**
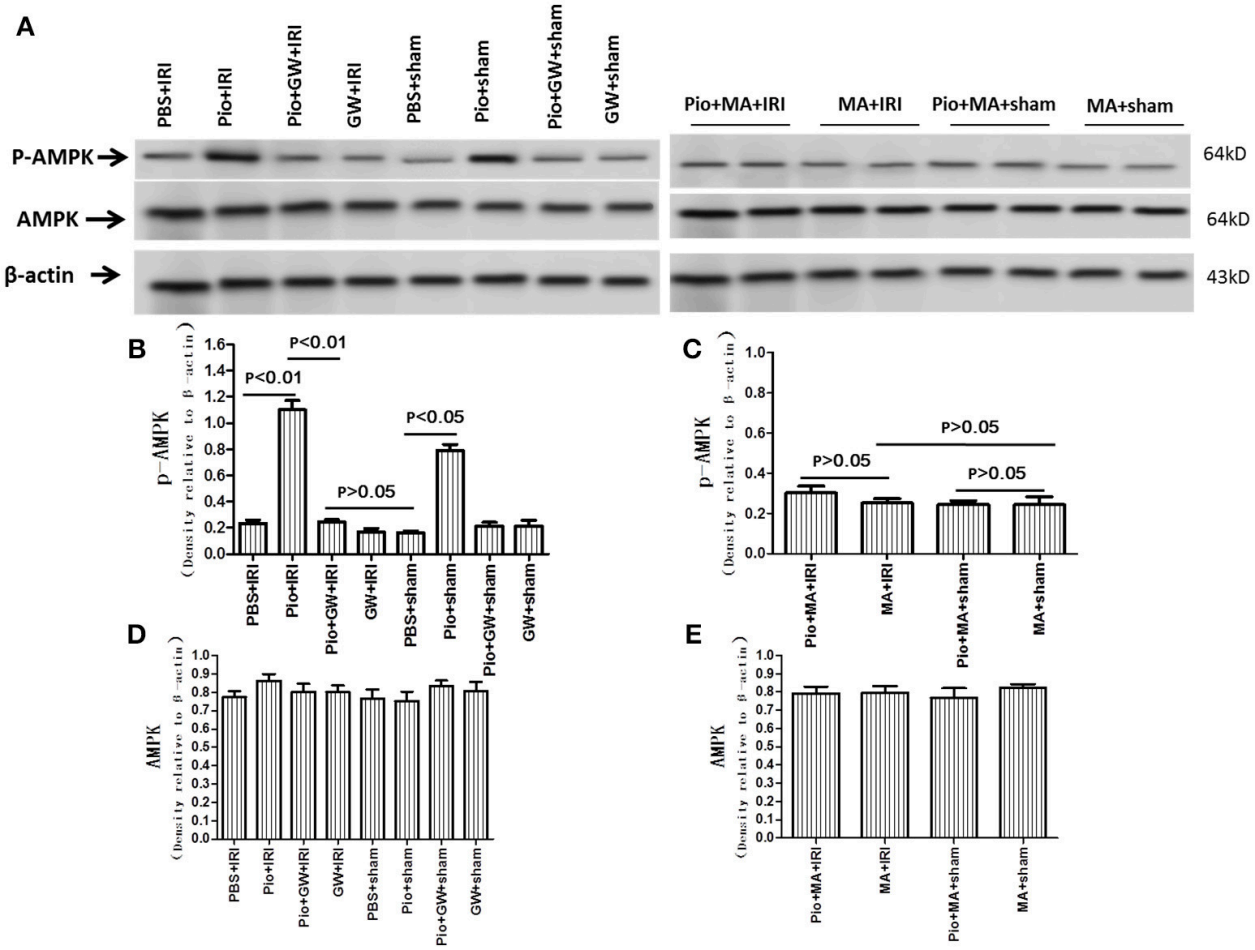
Expression of AMPK protein in renal tissue at 24 h after renal IRI. **(A)** Representative western blots show phosphorylated AMPK and total AMPK protein levels in the kidneys. **(B–E)** Quantitative analysis of phosphorylated AMPK protein and total AMPK protein levels in the kidneys. Values are expressed as means ± SE **(B–E)**. *n* = 6 in each group. AMPK, AMP-activated protein kinase. p-AMPK, phosphorylated AMPK.

To investigate the cellular mechanisms mediating the effects of Pio in IRI, we measured the total expression and phosphorylation level of AMPK in the kidneys by western blot. The expression of phosphorylated AMPK in the kidneys harvested from Pio-treated rats was significantly higher than that in PBS-treated rats; however, GW9662 and 3-MA suppressed this effect. In contrast, the total expression of AMPK in the kidneys remained unaltered (Figure 10).

### Pio decreased the expression of cleaved caspase-3 and caspase-8 proteins in renal tissue

**Figure 11:**
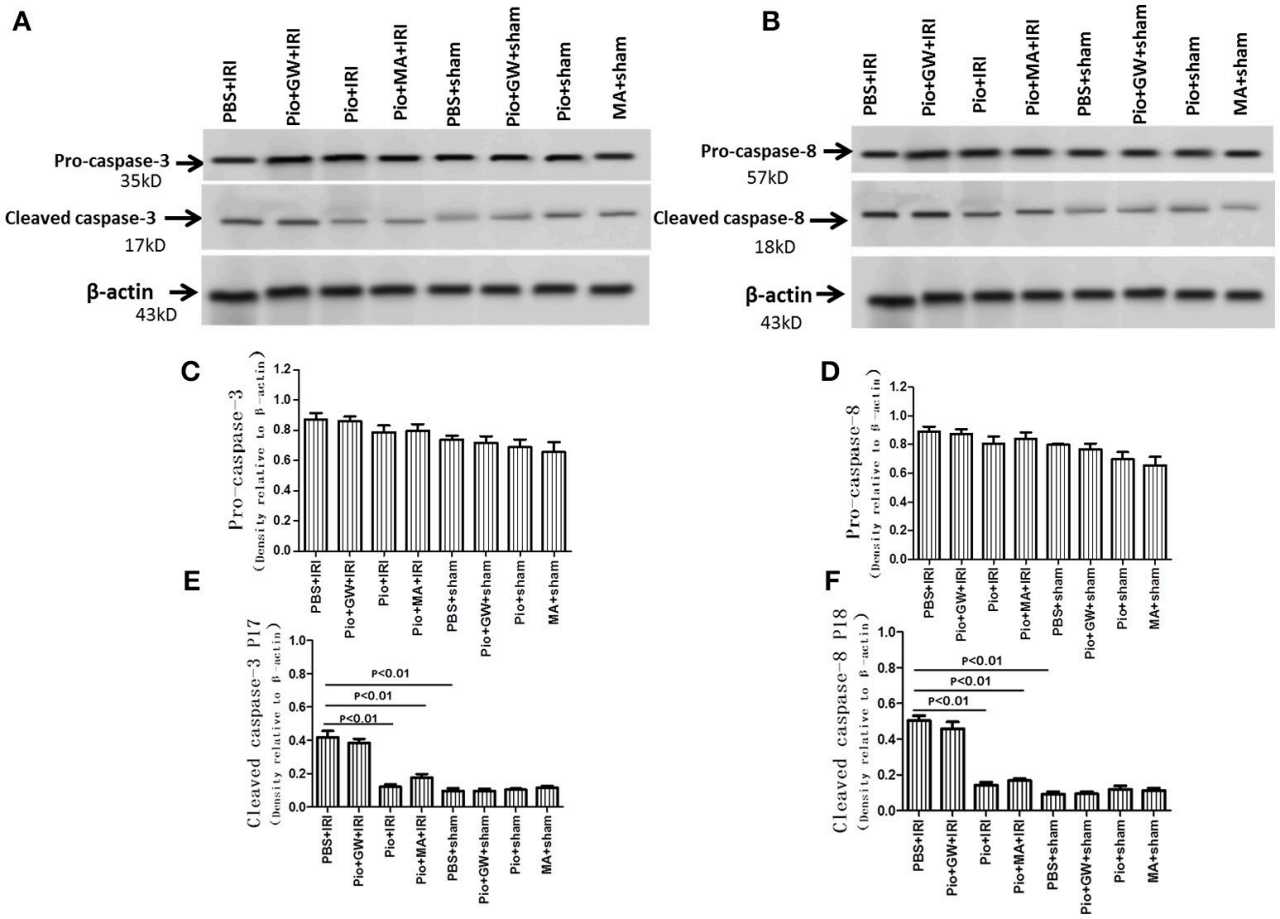
Expression of caspase-3 and caspase-8 protein in renal tissue at 24 h after renal IRI. **(A)** Representative western blots show pro-caspase-3 and cleaved caspase-3 P17 protein levels in the kidneys. **(B)** Representative western blots show pro-caspase-8 and cleaved caspase-8 P18 protein levels in the kidneys. **(C–F)** Quantitative analysis of caspase-3 and caspase-8 protein levels in the kidneys. Values are expressed as means ± SE **(C–F)**. *n* = 6 in each group.

Caspase-3 and caspase-8 are key-proteases involved in the caspase-dependent pathway of apoptosis. To ascertain the role of Pio in renal IRI, we examined the expression of caspase-3 and caspase-8 protein in the kidneys by western blotting at 24 h after reperfusion. No significant difference was noted between procaspase-3 and procaspase-8 protein expression among all groups (*P* > 0.05). However, the expression of caspase-8 P18 fragments and caspase-3 P17 fragments was significantly higher in rats subjected to IRI than in sham-operated rats (*P* < 0.01). The administration of Pio significantly decreased the level of caspase-8 P18 fragments and caspase-3 P17 fragments (*P* < 0.01) (Figure 11).

## Discussion

Our previous studies have shown that Pio, a PPAR-γ agonist used in the treatment of type 2 diabetes, could protect against renal IRI by inhibiting renal cell apoptosis and exhibiting an antioxidant effect (Hu et al., 2012; Zou et al., 2013). However, in the present study, we further demonstrated that Pio significantly attenuated kidney injury by activating autophagy in a rat model of renal IRI. We therefore investigated the mechanisms underlying the renoprotective effect of Pio and found that Pio improved all the histopathological and biological parameters tested in rats subjected to IRI, versus control rats.

First, Pio could reduce the serum levels of creatinine and urea nitrogen to improve the renal function of IRI rats. In addition to ameliorating the alterations in renal function parameters, Pio decreased the expression of some of the markers of acute renal injury such as KIM-1 and NGAL. KIM-1 has been shown to be strongly expressed and released by injured proximal tubular epithelial cells (TECs) (Lim et al., 2013), whereas NGAL is synthesized in the thick ascending limb of Henle's loop and collecting ducts (Mishra et al., 2003). Thus, the results of this study indicate that Pio ameliorated renal injury by affecting different parts of renal tubules.

Then, we also investigated the apoptosis of renal cells during this process. The inhibition of tubular cell apoptosis by Pio following IRI was in line with the results of previous studies that some pharmacological compounds decrease apoptosis and abrogate renal cell injury (Hu et al., 2012; Lee et al., 2017; Xu et al., 2017). For a deeper insight, TUNEL assay was performed to detect the effect of Pio on renal cell apoptosis in IRI rats. Consistent with the result of Reel et al. (2013), Pio could significantly reduce the rate of apoptosis, while the PPAR-γ inhibitor GW9662 and the autophagy inhibitor 3-MA inhibited the effect of Pio, further indicating that Pio could significantly reduce IRI-induced apoptosis.

Caspase signaling is a key molecular pathway involved in apoptosis in IRI. Our previous studies have documented caspase activation in the kidney, following IRI (Hu et al., 2014). In the present study, the administration of Pio decreased the levels of caspase-8 and caspase-3 cleavage in the kidney. Thus, Pio prevents the apoptosis of renal cells through inhibition of the apoptotic cascade at the mitochondrial level and at the levels of caspase-8 and−3. Li et al. (2016) observed the same phenomenon in rats subjected to renal IRI, where hydrogen-rich saline solution prevented acute kidney injury (AKI), upregulated bcl-2, down-regulated bax, and inhibited the activation of caspase-8, caspase-9, and caspase-3.

PPAR-γ is a nuclear transcription factor activated by corresponding ligands. Once activated by agonists, PPAR-γ regulates the transcription and expression of corresponding factors. In the present study, Pio significantly up-regulated the mRNA and protein expression of PPAR-γ. We postulated that Pio increased PPAR-γ transcriptional activity. Sato et al. (2013) reported that Pio activated PPAR-γ transcriptional activity in MCF-7 cells (human breast cancer cell line). Pio elevated luciferase reporter activity to a greater extent than that observed in the control sample that was transfected with PPRE and without any drug treatment. Thus, further detailed studies are needed for a better understanding of the transcriptional activity of PPAR-γ in TECs.

Interestingly, we found that the autophagy inhibitor 3-MA could alleviate the effect of Pio, further indicating that, in addition to apoptotic effect, cell autophagy may also participate in this process. Thus, we further investigated the mechanisms involved.

The execution of autophagy involves three critical proteins including LC3 II, p62, and Beclin-1 (Liang et al., 1999; Tanida et al., 2005; Bjørkøy et al., 2006). We found that autophagy was markedly enhanced as a result of higher expression of Beclin-1 and LC3-II in the kidneys treated with Pio. In contrast, the modest effect of the inhibition of autophagy by 3-MA on the induction of apoptosis suggests that other factors may also play an important role in IRI. Pio treatment also down-regulated p62 Furthermore, p62 is a key factor that controls cell death versus survival and is an autophagy-related protein normally degraded by lysosomal proteases through interaction with LC3II (Ichimura and Komatsu, 2010). Its accumulation reflects the inhibition of proteasomal activity (Zhang Y. B. et al., 2013). Similarly, diminished p62 level is associated with autophagy activation.

Autophagy plays a role to protect organisms against the pathogenesis of a disease, such as infections, cancer, neurodegeneration, and heart diseases (Parajuli and MacMillan-Crow, 2013). Autophagy induction has also been found to protect the kidney against cisplatin or IRI-induced acute injury (Lim et al., 2010). Parajuli and colleagues also suggested that mitochondrial biogenesis and autophagy played a protective role in IRI-induced acute oxidative stress and injury in the kidney (Parajuli and MacMillan-Crow, 2013). Autophagy-deficient mice exhibited significantly greater elevation in proximal tubule cell apoptosis and serum urea nitrogen and creatinine during renal IRI (Kimura et al., 2011). In the present study, we found that Pio decreased cell injury by up-regulating AMPK phosphorylation and activating autophagy in the kidneys of IRI model rats.

The molecular mechanisms responsible for the stimulation of autophagy by AMPK are now being unraveled in various cells. AMPK inactivates mTORC1, which normally suppresses autophagy when the nutrient supply is adequate. Recent evidence also indicated that AMPK directly phosphorylated Unc-51-like kinase (ULK) 1, the mammalian homolog of the yeast kinase autophagy-related 1 (Atg-1), which had a critical role in the induction of autophagy (Egan et al., 2011; Kim et al., 2011). AMPK was reported to phosphorylate ULK-1 on various sites, including Ser^467^, Ser^555^, Thr^574^, Ser^637^, Ser^317^, and Ser^777^ (Egan et al., 2011; Kim et al., 2011). Interestingly, loss of function of either AMPK or ULK-1 resulted in defective mitophagy (Egan et al., 2011). AMPK binds a complex of ULK-1 and mAtg101; this interaction was nutrition-dependent and was inhibited by mTOR phosphorylation of ULK-1 on Ser^757^ (Kim et al., 2011). Thus, there is a complicated interplay among AMPK, mTORC1, and ULK-1 in regulating autophagy and the specific molecular mechanisms through which AMPK regulates autophagy during IRI require additional investigation.

Hypoxia-inducible factor-1 alpha (HIF-1α) is a master gene switch for major adaptive responses to hypoxia. Many studies have demonstrated that HIF-1α is activated in AKI with or without ischemia and ameliorates AKI by improving hypoxia (Nangaku et al., 2013). Thus, HIF-1α is critical for the survival of TECs (Conde et al., 2012). Interestingly, AMPK activity was shown to be important for HIF-1 transcriptional activity under hypoxic conditions (Lee et al., 2003). There was a significant overlap between the actions and targets of HIF-1 and AMPK. AMPK activity was induced by hypoxia even when cellular ATP levels were not significantly depleted (Laderoute et al., 2006; Gusarova et al., 2009). Moreover, there is some emerging evidence on the interaction between these two signaling pathways in other organs. For instance, AMPK-mediated autophagy in chondrocytes was shown to be HIF-dependent, which was itself a stimulator of autophagy (Bohensky et al., 2010). Additionally, inhibition of protein synthesis by suppression of mTOR during hypoxia occurred via AMPK in the short-term and via HIF in the long-term (Hallows et al., 2010; Wheaton and Chandel, 2011). These studies suggest that AMPK and HIF may closely interact to show a coordinated cellular response to hypoxic stress by regulating both the supply and demand of ATP.

In conclusion, in this study, we demonstrate for the first time that AMPK and autophagy-related signals are involved in the Pio-induced renoprotection against IRI. These findings suggest that AMPK-activated autophagy plays an important role in the protective effect of Pio against renal IRI.

## Author contributions

WC, CZ, SZ, YH, ZY, and HH acquisition, analysis, and interpretation of data and drafting the paper. XX, RK, and HH study conception and design, analysis and interpretation of data, and drafting the paper.

### Conflict of interest statement

The authors declare that the research was conducted in the absence of any commercial or financial relationships that could be construed as a potential conflict of interest.
